# Efficiency, Selectivity, and Robustness of Nucleocytoplasmic Transport

**DOI:** 10.1371/journal.pcbi.0030125

**Published:** 2007-07-13

**Authors:** Anton Zilman, Stefano Di Talia, Brian T Chait, Michael P Rout, Marcelo O Magnasco

**Affiliations:** 1 Laboratory of Mathematical Physics, The Rockefeller University, New York, New York, United States of America; 2 Laboratory of Mass Spectrometry and Gaseous Ion Chemistry, The Rockefeller University, New York, New York, United States of America; 3 Laboratory of Cellular and Structural Biology, The Rockefeller University, New York, New York, United States of America; Vanderbilt University, United States of America

## Abstract

All materials enter or exit the cell nucleus through nuclear pore complexes (NPCs), efficient transport devices that combine high selectivity and throughput. NPC-associated proteins containing phenylalanine–glycine repeats (FG nups) have large, flexible, unstructured proteinaceous regions, and line the NPC. A central feature of NPC-mediated transport is the binding of cargo-carrying soluble transport factors to the unstructured regions of FG nups. Here, we model the dynamics of nucleocytoplasmic transport as diffusion in an effective potential resulting from the interaction of the transport factors with the flexible FG nups, using a minimal number of assumptions consistent with the most well-established structural and functional properties of NPC transport. We discuss how specific binding of transport factors to the FG nups facilitates transport, and how this binding and competition between transport factors and other macromolecules for binding sites and space inside the NPC accounts for the high selectivity of transport. We also account for why transport is relatively insensitive to changes in the number and distribution of FG nups in the NPC, providing an explanation for recent experiments where up to half the total mass of the FG nups has been deleted without abolishing transport. Our results suggest strategies for the creation of artificial nanomolecular sorting devices.

## Author Summary

The DNA at the heart of our cells is contained in the nucleus. This nucleus is surrounded by a barrier in which are buried gatekeepers, termed nuclear pore complexes (NPCs), which allow the quick and efficient passage of certain materials while excluding all others. It has long been known that materials must bind to the NPC to be transported across it, but how this binding translates into selective passage through the NPC has remained a mystery. Here we describe a theory to explain how the NPC works. Our theory accounts for the observed characteristics of NPC–mediated transport, and even suggests strategies for the creation of artificial nanomolecular sorting devices.

## Introduction

The contents of the eukaryotic nucleus are separated from the cytoplasm by the nuclear envelope. Nuclear pore complexes (NPCs) are large protein assemblies embedded in the nuclear envelope and are the sole means by which materials exchange across it. Water, ions, small macromolecules (<40 kDa) [1], and small neutral particles (diameter <5 nm) can diffuse unaided across the NPC [2], while larger macromolecules (and even many small macromolecules) will generally only be transported efficiently if they display a particular transport signal sequence, such as a nuclear localization signal (NLS) or nuclear export signal (NES). Macromolecular cargoes carrying these signal sequences bind cognate soluble transport factors that facilitate the passage of the resulting transport factor–cargo complexes through the NPC. The-best studied transport factors belong to a family of structurally related proteins, collectively termed β-karyopherins, although other transport factors can also mediate nuclear transport, particularly the export of mRNAs (reviewed in [1,3–6]). NPCs can pass cargoes up to 30 nm diameter (such as mRNA particles), at rates as high as several hundred macromolecules per second—each transport factor–cargo complex dwelling in the NPC for a time on the order of 10 ms [7,8].

Here we focus on karyopherin-mediated import, although our conclusions pertain to other types of nucleocytoplasmic transport as well, including mRNA export. During import, karyopherins bind cargoes in the cytoplasm via their nuclear localization signals. The karyopherin–cargo complexes then translocate through NPCs to the nucleoplasm, where the cargo is released from the karyopherin by RanGTP, which is maintained in its GTP-bound form by a nuclear factor, RanGEF. The high affinity of RanGTP binding for karyopherins allows it to displace cargoes from the karyopherins in the nucleus. Subsequently, karyopherins with bound RanGTP travel back through the NPC to the cytoplasm, where conversion of RanGTP to RanGDP is stimulated by the cytoplasmic factor RanGAP. The energy released by GTP hydrolysis is used to dissociate RanGDP from the karyopherins, which are then ready for the next cycle of transport. Importantly, this GTP hydrolysis is the only step in the process of nuclear import that requires an input of metabolic energy. Overall, the energy obtained from RanGTP hydrolysis is used to create a concentration gradient of karyopherin–cargo complexes between the cytoplasm and the nucleus, so that the process of actual translocation across the NPC occurs purely by diffusion [1,3–6,9–15].

**Figure 1:**
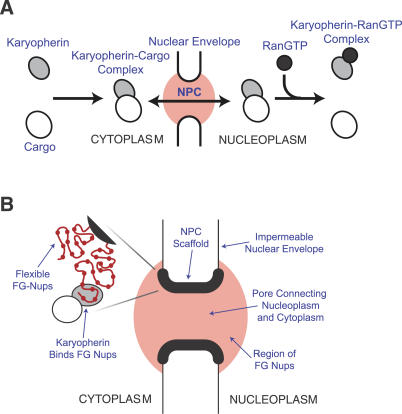
Main Features of the NPC and of Nuclear Import (A) Schematic of the nuclear import process. The karyopherins bind the cargo in the cytoplasm and transport it to the nucleus, where the cargo is released by RanGTP. (B) Schematic of the NPC. The nucleus and the cytoplasm are connected by a channel, which is filled with flexible, mobile filamentous proteins termed FG nups. The karyopherins carrying a cargo enter from the cytoplasm and hop between the binding sites on the FG nups until they either reach the nuclear side of the NPC or return to the cytoplasm.

Conceptually, nuclear import can be divided into three stages: first, the loading of cargo onto karyopherins in the cytoplasm, second, the translocation of karyopherin–cargo complexes through the NPC, and, third, the release of cargo inside the nucleus (Figure 1). The first and last stages have been the subject of numerous studies, and are relatively well understood, being soluble-phase reactions amenable to biochemical characterization (reviewed in [1,3–6,15]). The intermediate stage of transport is much less understood. Nevertheless, it is clear that the ability of karyopherins (and other transport factors) to bind a particular class of NPC-associated proteins containing phenylalanine–glycine (FG) repeats, known collectively as FG nups, is a key feature of the transport process, and allows them to selectively and efficiently pass with their cargoes through the NPC. In particular, experiments in which the FG nup–binding sites on the karyopherins were mutated show that disrupting the binding of karyopherins to FG nups impairs transport [6,12,13,15,16]. Current estimates of the binding affinity of karyopherins to most FG nups are in the range 1–1,000 nM (or 10–30 k_B_T per binding site), depending on the FG nup and karyopherin type [18–20]. Each FG nup usually carries a small region that anchors it to the body of the NPC, and a larger region characterized by multiple FG repeats. These FG repeat regions are natively disordered flexible chains or filaments that contain binding sites for transport factors (including karyopherins) and also appear to set up a barrier at the entrance of the NPC for macromolecules that cannot bind them [1,3,4,9,13,14,21–23]. The detailed physicochemical nature of this barrier is still under active study, although FG nups have been shown in vitro to form flexible polymer brushes when grafted to a surface [22] or gels in bulk solution [24]. Importantly, it has been repeatedly demonstrated that individual FG repeat regions can have a long reach, on the order of many tens of nm, within the NPC [3,22,23,25]. What is still needed is a quantitative theoretical explanation that can account for the observed characteristics of facilitated nuclear–cytoplasmic transport.

Here, we develop a diffusion-based theory to explain the mechanism of the intermediate stage of nucleocytoplasmic transport—i.e., translocation through the NPC. A useful theory of NPC-mediated transport should provide insight into several major unresolved questions, including: (i) How does the NPC achieve high transport efficiency of cargoes of variable sizes and in both directions, through only diffusion of the transport factor–cargo complexes? (ii) How does binding of transport factors to FG nups facilitate transport efficiency while maintaining a high throughput (up to hundreds of molecules per second per NPC) [7,8,12,13,26]? (iii) NPCs largely exclude nonspecific macromolecules in favor of transport factor–bound cargoes (reviewed in e.g., [4]). How is this high degree of selectivity achieved? (iv) Neither deletion of up to half the mass of the FG nups' filamentous unfolded regions, nor deletion of asymmetrically disposed FG nups' filamentous regions that potentially set up an affinity gradient, abolish transport [27]. Directionality of transport across the NPC can even be reversed by reversing the concentration gradient of RanGTP [28]. How can we account for such a high degree of robustness?

Several theoretical models have been proposed for the mechanism of transport through the NPC. These include the Brownian Affinity Gate model [4,14], Selective Phase models [12,13,29,30], the Oily Spaghetti model [1], Affinity Gradient models [10,11,15,20,31], the Dimensionality Reduction model [32], and most recently a Two-Gate model [48]. All these models can be thought of as viewing the NPC as a “Virtual Gate” [4,14], where the FG nups set up a barrier for entrance into the NPC and transport through the NPC involves facilitated diffusion controlled by association and disassociation of transport receptors with FG nups. They differ only in specific assumptions, such as the conformation and spatial deployment of the FG nups, their physicochemical state, or the distribution of affinities of binding sites (reviewed in [6]).

The aim of the present paper is to establish a general quantitative framework for NPC transport that is consistent with well-established structural and functional properties of the NPC and its components. We explain how the binding of karyopherins to the FG nups' flexible filaments inside the NPC can give rise to efficient transport. We demonstrate that competition for the limited space and binding sites within the NPC leads to a novel, highly selective filtering process. Finally, we explain how the flexibility of the FG nups could account for the high robustness of NPC-mediated transport with respect to structural changes [27]. We conclude by discussing verifiable experimental predictions of the model.

## Results

### Setting Up a Physical Model of NPC Transport

The NPC contains a central channel (approximately 35 nm in diameter) that connects the nucleoplasm with the cytoplasm. The internal volume of this channel, as well as large fractions of the nuclear and cytoplasmic surfaces of the NPC, is occupied by the flexible FG-repeat regions of the FG nups (i.e., that portion in each FG nup containing multiple FG repeats). Since these FG-repeat regions also protrude into the nucleus and the cytoplasm, the effective length of the NPC is estimated to be 70 nm [1,3,4]. The details of the distribution of the FG-repeat regions inside the central channel and the external surfaces of the NPC, as well as the exact number of binding sites on the karyopherins and the number of the FG-repeats on the FG-repeat regions that are accessible for binding, have not yet been well-established (although the number of the FG repeats is in the range of 5–50 per FG nup [6,27]). We made no specific assumptions about the distribution of FG nups, interactions between them, and their density, degree of flexibility, or conformation within the NPC. As we will discuss, the general features of transport through the NPC appear relatively insensitive to these details.

We represent transport through the NPC as a combination of two independent processes contributing to the movement of the karyopherin–cargo complexes through the central channel of the NPC: (i) the binding and unbinding of the karyopherins to the FG-repeat regions, and (ii) the spatial diffusion of the complexes, either in the unbound state or while still bound to a flexible FG-repeat region. The complexes entering the NPC from the cytoplasm thus stochastically hop back and forth inside the channel until they either reach the nuclear side, where the cargo is released by RanGTP, or return to the cytoplasm. Detachment from the FG-repeat regions and exit from the NPC can be either thermally activated, or catalyzed by RanGTP directly at the nuclear exit of the NPC [1,4]. A schematic illustration of transport through the NPC is shown in Figure 1.

### Enhanced Transport Efficiency Arises from the Karyopherins' Ability to Bind to FG nups

It is important to distinguish between two different properties of the transport process, namely, (i) the speed with which individual complexes traverse the NPC, and (ii) the probability that complexes, entering from the cytoplasm, arrive at the nuclear side [1,4,9,33–35]. As we discuss below, binding of karyopherins to the FG nups increases the probability of the karyopherins traversing the NPC, i.e., their transport efficiency; in the absence of such binding, the probability of traversing the NPC is low.

**Figure 2:**
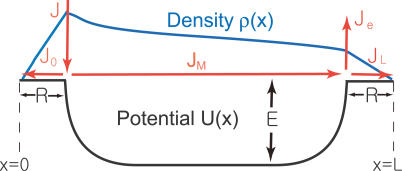
Transport through the NPC Is Modeled as Diffusion in an Energy Landscape The NPC channel is represented by a potential well U(x), shown in black. The complexes enter the NPC at x = R at an average rate J. A fraction of the entrance flux, J_M_, goes through to the nucleus. The rest return to the cytoplasm at an average rate, J_0_. The exit of the complexes from the channel into the nucleus occurs either due to thermal activation, with the rate J_L_, or by activated release by RanGTP, with the rate J_e_. Steady state particle density inside the channel, ρ(x), is shown in blue. It differs from what would be expected from equilibrium statistical mechanics as the complexes do not accumulate at the minimum of the potential but rather their density decreases toward the exit.

For simplicity, we assume that the unbinding and rebinding occur faster than the lateral diffusion of karyopherin–cargo complexes along the channel (although our conclusions were verified by computer simulations for any ratio of binding–unbinding rate to diffusion rate, unpublished data). In this limit, movement through the NPC can be approximated by diffusion in an effective potential as explained below. The strength of the effective potential depends on the relative strength of two effects. The first effect is the entropic repulsion between karyopherin–cargo complexes and FG-repeat regions and between the FG-repeat regions themselves, as the karyopherin–cargo complexes have to compress and displace the FG-repeat region filaments to enter the channel. The second effect is an attraction due to the binding of karyopherin–cargo complexes to the FG-repeat regions, as illustrated in Figure 2.

We represent the transport of karyopherin–cargo complexes through the NPC as diffusion in a one-dimensional potential, U(x) (expressed in units of k_B_T), in the interval 0 < x < L (Figure 2). The shape of the potential in the NPC is determined by the distribution of the FG nups along the channel (an issue we address later). The actual length of the NPC corresponds to the interval from x = R to x = L − R, and the regions of length R (on the order of the width of the channel [33,36]) at both ends of the interval correspond to the distance outside the NPC over which the particles diffuse into either the nucleoplasm or the cytoplasm. We did not directly model the diffusion of complexes outside of the NPC. Instead, we assumed that karyopherin–cargo complexes stochastically entered the NPC from the cytoplasm, with an average rate J at x = R, where J is proportional to the concentration of the karyopherin–cargo complexes in the cytoplasm [26,36]. Because of the random nature of movement inside the channel, a certain fraction of the complexes impinging on the channel entrance will not reach the nucleus and eventually return to the cytoplasm. We modeled this event by imposing absorbing boundary conditions at x = 0 and x = L that correspond to a karyopherin–cargo complex returning back to the cytoplasm, or going through to the nucleus, respectively.

Thus, the entrance current, J, splits into J_0_ and J_M_, corresponding to the flux of complexes returning to the cytoplasm and going through to the nucleus, respectively. Active release of the karyopherin–cargo complexes from the NPC by the nuclear RanGTP is modeled by imposing an additional exit flux, J_e_ (proportional to the nuclear concentration of RanGTP), at a position x = L − R. Therefore, the transmitted flux, J_M,_ splits into J_e_ and J_L_, which correspond respectively to the flux of karyopherin–cargo complexes released from the FG-repeat regions by RanGTP and to thermally activated release, as shown in Figure 2.

The efficiency of the transport through the NPC is determined by the fraction of the complexes that reach the nucleus, J_M_/J. We emphasize that we did not study the equilibrium thermodynamic properties of the channel, but rather the steady state, out-of-equilibrium behavior.

We neglected possible differences in the diffusion coefficient of the complexes inside and outside the NPC to focus on the role of karyopherin binding in the import process. We also assumed that no current enters the NPC from the nucleus as the cargoes are released from the karyopherins in the nucleus by RanGTP. Finally, we neglected variations of the potential in the direction perpendicular to the channel axis. The effects of these factors do not change our conclusions, and will be studied in detail elsewhere.

Under the above assumptions, the model can be solved using standard theory of stochastic processes [34]. Importantly, the model can be solved for a potential of an arbitrary shape, allowing us to model different distributions of binding sites within the NPC.

The transport of the karyopherin–cargo complexes through the NPC was then described by the diffusion equation for the density of complexes inside the channel, ρ(x)

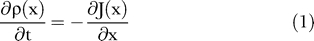
where the local flux of the complexes within the NPC, J(x), is given by:

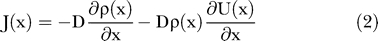
The first term in Equation 2 describes the random thermal motion of the complexes, and the second term stands for the variations in the flux due to local variations of the potential U(x); D is the diffusion coefficient of the complexes inside the channel.

The steady state density of the complexes in the channel, obtained by solving Equations 1 and 2, with entrance flux J and satisfying the boundary conditions ρ(0) = ρ(L) = 0, is

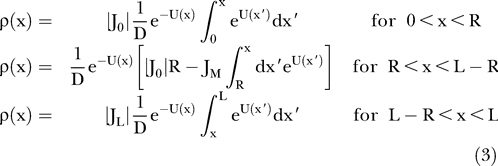



The sum of the flux of karyopherin–cargo complexes going through the NPC, and of that returning to the cytoplasm, is equal to the total flux of complexes entering the NPC; hence |J_0_| + J_M_ = J; similarly, J_M_ − J_L_ = J_e_. The flux, J_e_, is proportional to the number of complexes present at the nuclear exit, and to the frequency, J_ran_, with which RanGTP molecules hit the nuclear exit of the NPC: J_e_ = J_ran_ρ(x = L −R)R. Recalling that the potential outside the channel is zero (U(x) = 0) for 0 < x < R and L − R < x < L, and using the continuity of ρ(x) at x = L − R, one obtains for P_tr_, the probability of a given karyopherin–cargo complex reaching the nucleus (i.e., the fraction of complexes reaching the nucleus):

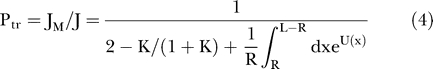
where K = J_ran_R^2^/D exp(−U(L−R)).

Equation 4 is the main result of this section and has several important consequences. The probability of traversing the NPC, P_tr_, defines the transport efficiency*.* This efficiency is seen to increase with the potential depth E, (defined as E = −min_x_U(x), Figure 2), proportional to the binding strength of the karyopherin–cargo complexes to the FG-repeat regions. In the absence of binding, P_tr_ is small (∼R/L), so that a complex will, on average, return to the cytoplasm soon after entering the NPC. Notably, an attractive potential inside the NPC increases the time the complex spends inside the NPC and thus increases the probability that it reaches the nuclear side, rather than returns to the cytoplasm.

When RanGTP only releases cargo from its karyopherin, but not from the FG-repeat regions (i.e., J_e_ = 0); the maximal translocation probability, P_tr_, is 0.5. However, in the case when RanGTP also releases karyopherin–cargo complexes from FG-repeat regions, the translocation probability, P_tr_, can reach unity. Importantly, the latter effect is more pronounced for a large K, that is, for strong binding at the exit. We shall discuss the practical implications of this result later.

The second important consequence of Equation 4 is that P_tr_ depends only weakly on the shape of the potential, U(x). This can account for why the transport properties of the NPC are relatively insensitive to the details of the distribution of FG-repeat regions inside the NPC, and to the distribution of the binding sites on the FG-repeat regions.

### A Mechanism for Selectivity is Provided by the Limited Space and Binding Capacity within the NPC

The previous section does not take into account the interference between karyopherin–cargo complexes inside the channel. Although a large interaction strength, E, increases transport efficiency, this increase is at the expense of an increased transport time T(E), which grows roughly exponentially with E (Text S1), and leads to an accumulation of karyopherin–cargo complexes inside the channel. However, the space and the number of available binding sites inside the channel are limited. As the number of these complexes in the channel increases, they start to interfere with the passage of each other due to molecular crowding. This molecular crowding results from two different sources. One is the repulsion that the entering macromolecules feel from the FG nups that set up the permeability barrier. The second is competition for the limited space inside the channel between the karyopherin–cargo complexes themselves, and which we now demonstrate can determine the selectivity. In effect, these two factors represent the entropic exclusion that we have discussed previously [1,3,4,9,13,14,21–23].

To quantitatively investigate how mutual interference between translocating karyopherin–cargo complexes and molecular crowding affect transport efficiency, we performed dynamic Monte Carlo simulations of the diffusion of complexes inside the NPC, in the potential U(x), using a variant of the Gillespie algorithm [37–39]. The simulations are a discrete version of the continuum formulation of the previous section. The interval [0,L] is represented by N discrete positions, which is a standard way to approximate the continuous diffusion; it is important to appreciate that these sites do not represent the actual binding sites, but correspond to the length of a diffusion step. We allowed only a limited number, n_max_, of complexes at each position at any moment of time, which models the competition between complexes for the limited space and the accessible binding sites inside the channel. In line with our analytical model above, karyopherin–cargo complexes were deposited at the position i_R_ if it was unoccupied by a complex, with a probability of JL^2^/(DN^2^) per simulation step. When a complex reached position i = 0 (cytoplasm) or i = N (nucleus), it was removed from the channel. In addition, the complexes present at the position i = N − i_R_ could be removed directly, with the probability J_ran_L^2^/(DN^2^), which models the effect of the release of the complexes from FG-repeat regions by nuclear RanGTP. Once inside the channel, a complex present at site i could hop to an adjacent unoccupied site, i ± 1, with the following probability:

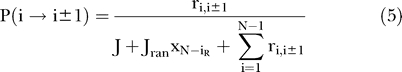
where x_i_ is the site occupancy: x_i_ = 0 if the site is unoccupied, x_i_ = n if n complexes are present at the position i, up to the n_max_ complexes. The transition rates from a site i to a site i ± 1 were 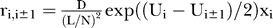 , if x_i±1_ < n_max_, and zero, if x_i±1_ = n_max_ [37–39].

**Figure 3:**
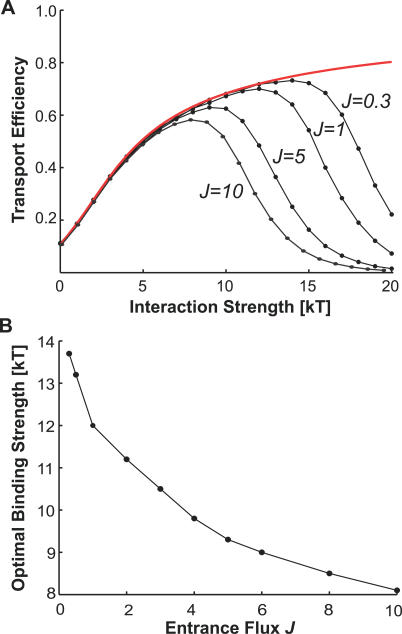
Transport Efficiency Is Determined by the Interaction Strength (A) Transport efficiency, as given by the probability to reach the nucleus, is shown as a function of the interaction strength. RanGTP activity in the nucleus is represented by using J_ran_L^2^/(N^2^D) = 1.5. The curves correspond to four different values of the entrance rate, J (measured in units of 10^−4^16D/R^2^); the red line is the low-rate limit of Equation 4. For any entrance rate, the transport efficiency is maximal at a specific value of the interaction strength, which provides a mechanism of selectivity. (B) Optimal interaction strength of (A) as a function of the incoming rate J (in units of 10^−4^16D/R^2^), for J_ran_L^2^/(N^2^D) = 1.5; parabolic potential shape. See Discussion for the corresponding actual values of the flux through the pore. Black dots are simulation results.

The results of our simulations for the experimentally relevant range of interaction strength E and incoming flux J are shown in Figure 3 (see Discussion for an explanation of our choice for the parameter values). Figure 3 shows the results for n_max_ = 1; the results did not change substantially for higher allowed local occupancy (Text S1 and Figure S5). For low interaction strength, E, the translocation probability curves for all entrance fluxes, J, collapse onto a single line (which is predicted by the analytical solution from the previous section), because in this regime there are few complexes simultaneously present in the channel, and molecular crowding is negligible. For stronger binding, karyopherin–cargo complexes accumulate inside the channel, blocking the inflow of additional complexes, and the channel becomes jammed as reflected in the decrease of the probability to traverse the NPC.

The main conclusion of the simulations, as shown in Figure 3A, is that transport through the NPC is maximal for an optimal value of the interaction strength E_c_. Therefore, our theory rigorously confirms the intuitive notion of the existence of an optimal binding that balances increased transport probability with increased time spent within the NPC. In our simulations, the optimal binding strength depends on the entrance flux, and thus on the abundance of complexes of a particular type in the cytoplasm. In particular, the optimal interaction strength is higher for low entrance fluxes, as illustrated in Figure 3B.

Existence of an optimal interaction strength provides a mechanism for the selectivity of NPC-mediated transport. Karyopherin–cargo complexes tuned for a particular strength of interaction with FG-repeat regions have a high translocation probability, while macromolecules that do not interact with FG nups are less likely to cross. We elaborate on this finding in the next section.

### Competition between Non-Specifically Binding Macromolecules and Karyopherins Enhances the Selectivity of the NPC

As discussed in the previous section, the binding of karyopherins to FG-repeat regions provides a mechanism of selectivity. However, the maximum depicted in Figure 3A is broad; the translocation probability is significant even for binding strengths considerably lower than the optimal one. For instance, if the optimal interaction strength is E = 15kT (Kd ≈ 300 nM), macromolecules whose interaction strength is 7kT (Kd ≈ 1 mM) [17] have a probability of reaching the nucleus that is more than half the optimal one. On one hand, this broad maximum allows NPC-mediated import to function efficiently across a broad range of transport factor binding strengths. On the other hand, this might also permit passage of macromolecules that bind nonspecifically to FG-repeat regions (e.g., due to electrostatic interactions). However, proper functioning of living cells requires a high selectivity of the NPC—how might this be achieved? So far, Figure 3 only takes into account the competition between complexes of identical binding strength for space inside the channel. However, in a situation where optimally binding karyopherins compete for space and binding sites inside the channel with other, weakly binding macromolecules, the passage of the latter is sharply reduced, which significantly increases the selectivity of the NPC. Qualitatively, because the strongly binding karyopherins and karyopherin–cargo complexes spend more time in the NPC, a weakly binding macromolecule entering the channel will—with high probability—find it occupied by karyopherin–cargo complexes. Therefore, because the residence time of a low affinity macromolecule is relatively short, there is a high probability that it will return to the cytoplasm before the channel clears. On the other hand, if a karyopherin–cargo complex enters a channel that is already occupied by other strongly binding complexes, there is still a high probability that, due to its relatively high residence time, it will reside inside the channel long enough for the complexes that are already inside the NPC to get through. As free karyopherins exchange back and forth across the NPC constantly, there will always be karyopherins (or other transport factors) binding in the NPC and so excluding nonspecific macromolecules, making the NPC a remarkably efficient filter.

These heuristic arguments were verified via computer simulations, using the algorithm of the previous section, adapted to account for two species of particles of different binding strengths. Two species of particles of different binding affinities (representing a karyopherin–cargo complex and another macromolecule that can bind nonspecifically (and weakly) to the FG-repeat regions), are deposited stochastically at the NPC entrance with the same average rate J. As in the previous section, the particles diffuse inside the channel until they either reach the nucleus, or return to the cytoplasm. Due to limited space, each position can be occupied by only a limited number of particles (see Text S2 for the actual code).

**Figure 4:**
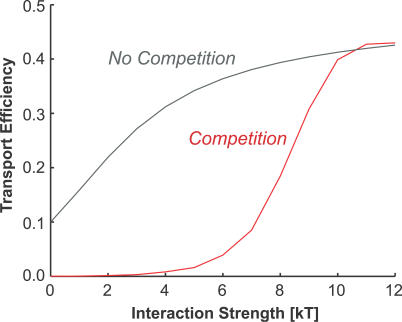
Karyopherins Efficiently Exclude Nonspecifically Binding Macromolecules from the NPC Shown is the transport efficiency of particles across the NPC as a function of interaction strength with the FG-repeat regions, either in the presence or absence of competing particles. Gray line: transport efficiency of particles as a function of interaction strength in the absence of competition. Red line: transport efficiency of a weakly binding species in an equal mixture of weakly and strongly binding species, as a function of the interaction strength of the weakly binding species; the interaction strength for the strongly binding species is 12k_B_T. Translocation of the weakly binding species is sharply reduced in the presence of the strongly binding species, until its binding strength approaches that of the strongly binding species. No RanGTP activity was included in these simulations, hence lowering the transport efficiency compared with Figure 3.

As Figure 4 shows, competition for the space inside the channel between the translocating particles dramatically narrows the selectivity curve as compared with Figure 3. This effect is a novel mechanism for the enhancement of transport selectivity beyond what is expected from the equilibrium binding affinity differences alone. In contrast to other mechanisms of specificity enhancement (e.g., kinetic proofreading [40]), no additional metabolic energy is required for this enhanced discrimination. Instead, selectivity is achieved by competition producing a differential NPC response to two ranges of binding affinities. There remains a broad range of higher binding affinities, occupied by transport factors, wherein passage across the NPC is efficient; however, in the low range of affinities, transmission is effectively prevented. We emphasize that this is an essentially nonequilibrium effect, and the selectivity enhancement goes far beyond the difference in the equilibrium binding affinities. Importantly, the enhancement of the selectivity persists even when high local occupancies are allowed (Text S1 and Figure S4).

### The Flexibility of FG-Repeat Regions May Account for the Robustness of NPC-Mediated Transport

In the previous sections, we used a continuous potential to model transport through the NPC. However, in reality the translocating karyopherin–cargo complexes likely hop between discrete binding sites that are located on the separate (or on the same) flexible FG-repeat regions, which fluctuate in space around their anchor points due to thermal motion [3,22,23]. This flexibility allows the complexes to diffuse along the channel while remaining bound to an FG-repeat region. A complex can also unbind from an FG-repeat region and rebind again to the same or a neighboring FG-repeat region, moving while unbound by passive diffusion. In this section, we elucidate how the number of FG nups inside the channel affects transport.

**Figure 5:**
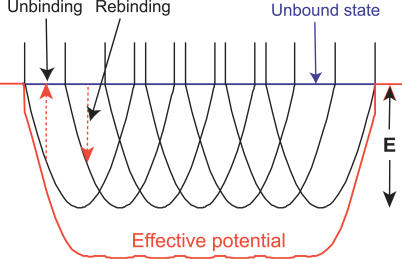
Discrete Overlapping FG-Repeat Regions Can Be Approximated by a Smooth Effective Potential Transport through the NPC can be represented as diffusion in an array of potential wells (solid black lines) that represent flexible FG-repeat regions whose fluctuation regions overlap. The red dotted arrows correspond to the complexes unbinding from and rebinding to the FG-repeat regions. The solid blue line represents the unbound state. The solid red line shows the equivalent potential in the case when the unbinding of the complexes from the FG-repeat regions is much faster than the lateral diffusion across an individual well.

Under these assumptions, the translocation of karyopherin–cargo complexes through the NPC can be described as diffusion in an array of potentials, as illustrated in Figure 5, where each potential well U_i_(x) of a width p_i_ represents an FG-repeat region. The shape of each well depends on the number of the binding sites on each FG-repeat region, the binding strength of the karyopherin–cargo complex, and the rigidity and the length of an FG-repeat region, which determine the cost of its entropic stretching in the process of spatial fluctuations. This description allows for the possibility of having several binding sites on an FG nup which affects only the shape of the wells (Text S1 and Figure S1). The blue line in Figure 5 corresponds to the unbound state. Although for the purposes of illustration all the wells are shown to have the same form, the subsequent results are valid for an arbitrary distribution of potential shapes. We shall denote the density of the karyopherin–cargo complexes in the i-th well as ρ_i_(x) and the density of unbound complexes as ρ_0_(x). The lateral diffusion of the complexes, combined with the binding and unbinding to the FG-repeat regions is then described by the following equations [41]:

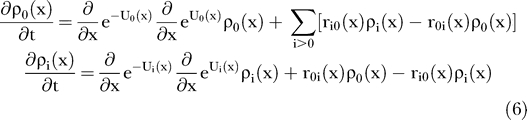



The first term in the first equation describes the diffusion in the unbound state, while the second and the third terms describe the unbinding and the binding to the wells. Similarly, the second equation describes the diffusion while still bound to the i-th well (FG-repeat region). The local unbinding and binding rates from the i-th well are r_i0_(x) and r_0i_(x), respectively. They are related by the detailed balance condition, *r*_0*i*_(*x*)/*r*_*i*0_(*x*) = *e* ^−*U*_*i*_^ ^(*x*)+*U*_0_(*x*)^, which reflects the energy difference between the bound and unbound states. If the unbinding rates are fast compared with the diffusion time across the wells, the relative densities of bound and unbound complexes are at their local equilibrium Boltzmann ratio [41]: 
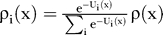
 , where ρ(x) = ∑_i_ρ_i_(x) is the total density of the complexes at a position x. Adding up Equations 6, one obtains an equation for the total density of the complexes at position x, ρ(x):

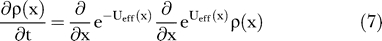
where 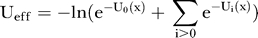 [41]. Thus, the process of translocation through an array of flexible FG nups within the NPC can be described as simple lateral diffusion in the effective potential U_eff_, which leads to Equations 1 and 2 in the first section of this manuscript, which are essentially identical to Equation 7, even though they are written in a slightly different form.

As we have proposed in previous sections, the transport properties of the NPC are relatively insensitive to the detailed shape of the effective potential. It follows from Equations 6 and 7 that the shape of the effective potential depends only weakly on the number and the shape of the overlapping potential wells corresponding to the FG-repeat regions.

**Figure 6:**
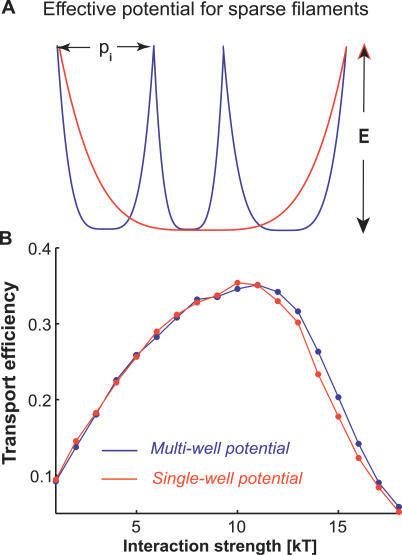
The Number of FG nups Does Not Significantly Affect the Transport Properties of NPCs (A) Effective potential for sparse flexible FG-repeat regions is shown as a blue line. Each well corresponds to an FG-repeat region. The transport properties in this multiwell potential are independent of the number of wells, and hence equivalent to those in the single-well potential, shown as a red line. (B) Numerical simulations show essentially identical transport efficiencies in the multiwell potential (blue line) and in the single-well potential (red line).

Even more strikingly, in our model the transport properties of the NPC are not very sensitive to the number of the FG-repeat regions. This is robust with respect to the variations in the number of the FG-repeat regions [27]. This was in striking contrast to the case of the inflexible FG-repeat regions when the binding sites are sparsely distributed without a large degree of overlap (Text S1 and Figure S2). We illustrate this point through a limiting case where the FG-repeat regions barely touch, represented by the potential shown by the blue line in Figure 6A. The flat central part of each potential well corresponds to where the karyopherin–cargo complex is diffusing in the channel while bound to an FG-repeat region, and the sharply rising regions at the borders correspond to unbinding of the complex and its transfer to the next filament. Narrow wells correspond to filaments with limited reach, while wide wells correspond to filaments that can stretch a long distance without significant entropic cost. The potential wells can have different widths, p_i,_ so that their combined width is equal to the total length of the channel, 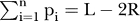 . All the potential wells have the same shape, U_0_, re-scaled to the width of an individual well, so that the potential at a point x = ∑_j<i_p_j_ + Δx is: U(x) = U_0_(Δx/p_i_).

Crucially, the transport properties of the potential shown in Figure 6A do not depend on the number of wells. Both the translocation probability and the residence time are equivalent for the multiwell potential shown in blue, and the single-well potential shown in red, obtained by rescaling an individual blue well to the whole length, L − 2R, of the channel. Indeed, it follows from Equation 4 that the translocation probability for the multiwell potential with *n* wells, is

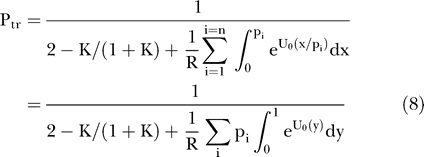
which is independent of the number and the width of the wells because ∑_i_p_i_ = L − 2R; as before, K = J_ran_R^2^/D exp(−U(L − R)). We prove in Text S1 that the residence time is similarly independent of the number of wells. Since both translocation probability and residence time are independent of the number of wells, the transport properties do not depend on the number of wells, even for high entrance rates or binding strengths, when jamming becomes important, as verified by computer simulation (Figure 6B).

This result highlights the robustness of our model of NPC transport; in multiwell potentials of this type, the NPC's transport properties do not depend on the specific number of FG-repeat regions, so long as they are flexible enough for their fluctuation regions to overlap, permitting complexes to freely transfer from one filament to the next, which might explain the puzzling degree of robustness of the NPC transport with respect to the deletion of FG repeat regions [27].

## Discussion

Several conceptual models have been proposed to describe transport through the NPC [1,3–6,9–15,29–32,48]. Most propose that this transport relies on diffusion of the transport factor–cargo complexes in the environment of flexible FG-repeat regions of the FG nups, controlled by transient binding to the FG nups these flexible regions [1,3–6,9–11,14–16].

We have formulated and solved a rigorous mathematical model of transport through the NPC that depends on the physics of diffusion in a channel combined with binding to the flexible filamentous FG-repeat regions (without making detailed assumptions about the conformation and distribution of FG-repeat regions inside the channel). Our model applies to both export and import processes and explains the main features of NPC-mediated transport; namely, its high selectivity for cargoes bound to transport factors, its efficiency and directionality, and its robustness to perturbations.

We propose that the selectivity of the NPC arises from a balance between the probability (efficiency) and the speed of transport of individual karyopherin–cargo complexes. Analogous ideas have been suggested to account for the transport properties of ion channels and porins [14,33–35,42]. In our model, the probability of a karyopherin–cargo complex reaching the nucleus increases with the binding strength of the transport factors to FG-repeat regions, but at the expense of increased residence time inside the NPC; eventually, complexes spend so much time in the NPC that they impede the passage of other complexes through the channel. Therefore, there is an optimal value of the binding strength of karyopherins to FG-repeat regions that maximizes their transport efficiency through the NPC. For karyopherin–cargo complexes with lower entrance fluxes, the optimal binding strength is higher because at low fluxes the accumulating complexes can reside longer in the channel without blocking it (Figure 3). This correlation of optimal binding strength and entrance flux could explain why there are different karyopherin types; the binding strength of each karyopherin type might be related to its cargoes' relative flux and hence the abundance of its cargoes.

By considering known parameters of the nuclear transport machinery, we can test whether our simulations are consistent with the experimentally observed values of the flux through the NPC, and the residence time inside it. We take the effective length of the NPC as L ∼ 70 nm and its effective passive diffusion diameter as R ∼ 7 nm, within the range observed for different NPCs [1]. The diffusion coefficient D of the complexes inside the channel can be estimated to lie in the range 1–10 μ^2^/s, typical for protein diffusion in the crowded environment of the cytoplasm [43,44]. In vivo, the total cargo flux through an NPC ranges from several molecules per second up to several hundred molecules per second [12,26,45–47]. Accordingly, we performed simulations for values of the incoming flux J in the range 0.3–10 in units of (10^−4^16D/R^2^), which corresponds to a flux through the NPC in the range of 10–1,000 molecules per second. This results in predicted residence times in the NPC (given by LR/Dexp(E/kT)) of approximately 0.01–1 s, consistent with experimentally determined residence times [7,8]. For incoming flux values in this range, our model predicts optimal interaction strengths in the range of 5–15k_B_T (Figure 3). Molecular dynamics calculations predict higher binding energies [18], but the effective interaction strength in our model is reduced by entropic effects from the flexible FG-repeat regions.

Our model can also explain the high specificity of facilitated transport through the NPC, wherein each NPC permits the passage of transport factor–cargo complexes but efficiently filters out macromolecules that do not bind specifically to the FG-repeat regions. The difference in binding energy between specifically and nonspecifically binding macromolecules can be as little as a few k_B_T, which may not seem enough for such efficient discrimination. However, we have uncovered an additional mechanism that we believe significantly enhances the specificity of NPC transport. This mechanism relies on the direct competition between transport factors and nonspecifically binding macromolecules; they compete for space and binding sites in the channel. As a consequence of their stronger binding, transport factors have a longer residence time within the channel as compared with nonspecifically binding macromolecules, which are therefore outcompeted for space and binding sites within the channel. The constant flux of cargo bound or free transport factors between the nucleus and cytoplasm therefore effectively excludes nonspecifically binding macromolecules from the channel. We emphasize that this selectivity enhancement is essentially a nonequilibrium kinetic effect. Hence, although no metabolic energy is expended in this filtering process [40], the resulting selectivity is much higher than might be expected from just the different binding affinities of transport factors and nonspecific macromolecules (Figure 4).

In the case of karyopherin-mediated import, the transport efficiency is enhanced when RanGTP directly releases karyopherins from their binding sites on FG-repeat regions at the NPC exit [1,4,20,31]—an enhancement that increases with the binding strength at the nuclear exit. High affinity binding sites at the nuclear exit of the NPC decrease the probability of return, once a complex has reached the nuclear side. This result may account for the observed high affinity binding sites that are localized at the nuclear side on the NPC in import pathways and at the cytoplasmic side in export pathways [1,4,20,31].

Although the transport properties of the NPC depend strongly on the magnitude of the interaction strengths between transport factors and FG-repeat regions, we predict that transport depends only weakly on spatial variations of the binding strength along the channel. In particular, a gradient of binding affinity across the NPC should not, by itself, increase throughput compared with a uniform distribution of the same sites. This could explain how transport can be reversed across the NPC simply by reversing the gradient of RanGTP [28]. Only a high affinity trap at the exit of the NPC in combination with the action of RanGTP in releasing the karyopherins from this trap can improve the throughput through the NPC.

Although transport relies on the flexibility of the FG-repeat regions, it is relatively insensitive to the number of flexible FG-repeat regions inside the NPC—as long as their fluctuation regions can overlap (Figures 5 and 6). This could account for recent experiments in which up to half the total mass of the flexible FG-repeat regions in NPCs were deleted without abrogating nucleocytoplasmic transport [27]. In particular, it follows from Equation 4 that the probability of traversing the NPC is low if the binding sites are sparse and stationary, unless they are so dense that they occupy almost all the available length of the channel. However, in this case, transport is sensitive to the number of sites. Thus, a theory that neglects the flexibility of the FG nups is incapable of explaining how the NPC can sustain a high throughput and be relatively insensitive to the removal of up to half the binding sites. Importantly, this result does not depend on the speed of the diffusion of the karyopherin–cargo complexes between the binding sites. By contrast, a model that relies on stationary binding sites predicts that transport will not be robust to deletion of half the binding sites (Figures S2 and S3). Hence, we show that not every diffusion-based mechanism can explain the robustness of transport with respect to deletion of the FG-nups. Moreover, different karyopherins can bind different specific FG-nups. Thus, they can follow different pathways within the NPC channel, each reliant on a small subset of specific FG-nups [27]; we thus predict that only removal of this small subset would prevent that karyopherin from transiting the NPC. One of the conclusions drawn by [27] from their results is that the lethal deletions have removed all the preferred FG-binding sites on an essential pathway; our model is thus completely in line with their work.

Experimental tests for our model's predictions include varying the effective potential experienced by transport factor–cargo complexes inside the NPC by systematically introducing mutations into the binding sites [16], changing the cargo size [2], or using cells with genetically modified numbers of the FG-repeat regions [27]. Finally, any device built according to the principles outlined above would possess the transport properties described by our model, suggesting strategies for the creation of highly selective artificial nanomolecular sieves.

## Materials and Methods

The simulations were written in C language and run on a cluster of UNIX processors. The simulation algorithm is described in the text; see Text S2 for the actual code. Analytical calculations were in part performed with pencil and paper, or in some cases using Mathematica version 5.1.

## Supporting Information

Figure S1 Several Binding Sites on a Single FG-Repeat Region Result in an Effective Potential (492 KB EPS)

Figure S2 Array of Discrete Bindings Sites (381 KB EPS)

Figure S3 Transport Efficiency in the Case of Stationary Binding Sites (902 KB EPS)

Figure S4 Karyopherins Efficiently Exclude Nonspecifically Binding Macromolecules from the NPC, Irrespective of the Permitted Local Occupancies (590 KB EPS)

Figure S5 Translocation Probability for High Local Occupancy (1.4 MB EPS)

Text S1 Additional Information (422 KB DOC)

Text S2 Simulation Code for the Diffusion of Complexes through the NPC Channel (61 KB DOC)

## Abbreviations

FG repeats: phenylalanine–glycine repeats
FG nups: a class of NPC-associated proteins containing FG repeats
NPC: nuclear pore complex

## Note Added in Proof

Reference [48] is cited out of order in the article because it was added while the article was in proof.
