# Supplementary material for: Efficiency, Selectivity, and Robustness of Nucleocytoplasmic Transport
Source: PLoS Comput Biol. 2007 Jul 13;3(7):e125. doi: 10.1371/journal.pcbi.0030125 (PMC1914370; doi:10.1371/journal.pcbi.0030125)
Supplement: Supplementary file 1 [file pcbi.0030125.sd001.doc]

**Supplementary text 1: Additional Information**

**Residence time in a multi-well potential**

Here we calculate the residence time in the potential of Fig. 5. As before, we assume that the potential is zero in the intervals $[\text{0,}R]$ and $[L-R,L]$. The particles start their diffusion from $x=R$. For an arbitrary distribution of wells widths $p_{i}$, the potential is equal to $U_{0}(x/{p_{i}})$ in an interval $x_{i}<x<x_{i+1}$, where $p_{j}$. The mean first passage time, until a particle either reaches to $x=L$ or returns to $x\text{=0}$ (with $J_{e}\text{=0}$), is:

$T(U)/D=P_{\text{tr}}(U)T_{\to}(U)+(1-P_{\text{tr}}(U))T_{\leftarrow}(U)$	S

where $y\text{dze}^{-U(z)}$; $R\text{dze}^{-U(z)}$ and $Le^{U(x)}$ is the translocation probability (Gardiner, 2004).

Thus, $T_{\leftarrow}=R^{2}\text{/2}$ and

$ye^{-U(x)}\text{dx}$	S

The second and the third terms in the above expression become, respectively:

$i-1p_{i}p_{j})I_{0} = p_{0}^{2}I_{0}$	S

where $1\text{dxdye}^{U_{0}(x)}e^{-U_{0}(y)}$. Thus, the mean residence time $T(U)$ does not depend on the number of wells $n$.

The effect of the additional exit current $J_{e}$ at $x=L-R$ is formally equivalent to shortening the interval $[L-R,L]$ to $[L-R/(1+K),L]$. Repeating the calculation above for this case shows that also for non-zero $J_{e}$ the residence time does not depend on the number of wells.

**Dependence of the residence time on the binding strength**

Here we derive a simple expression for the dependence of the residence time $T$ on the interaction strength $E$, by assuming that the interaction strength is constant throughout the channel. In this case, using the equation S, one obtains the expression:

$T=\frac{\text{RL}}{2}(\text{exp}(E/{k_{B}}T)(1-R/L)+R/L)$	S

which varies roughly exponentially for large values of $E/{k_{B}}T$.

**Effective potential for multiple binding sites on an FG-repeat region.**

Each FG-repeat region bears several bindings sites, ranging from 5 to 30 (Strawn et al., 2004). It is not clear how many of those are accessible for binding simultaneously, because they can be partially occluded by the chains. Nevertheless, our model accounts for multiple binding sites on a single FG-repeat region. Analogously to the derivation in Section 'The flexibility of FG-repeat regions accounts for the robustness of NPC-mediated transport' above, the movement of a karypherin-cargo complex along multiple binding sites that are located on the same flexible chain, can be represented by diffusion in an array of potentials shown in Fig. S1. The black wells correspond to the diffusion of a complex while it is bound to a given binding site. The width of each well is determined by the distance of the binding site from the anchor point of the FG nup. Using equation (6) from the paper, the movement of a complex reduces to the diffusion in the effective potential shown in red line.

**Transport properties in an array of discrete and stationary binding sites**

In this section we discuss the transport in the channel that contains *discrete and stationary* binding sites, shown in Fig. S2. This picture corresponds to the situation where the FG-repeat regions are not flexible, and are not free to fluctuate around their anchor points. Using equation (4) from the paper, one gets for the translocation probability in the case of $n$ binding sites of width $a$ and strength $E$:

$P_{\text{tr}}=\frac{1}{L/R-\text{na}/R(1-\text{exp}(-E))}$

where, as above, $L$ is the length of the channel and $R$ is its radius. The behavior of the transport efficiency $P_{\text{tr}}$ is shown in Fig. S3 A where it is plotted as a function of the number of the binding sites $n$, for $a\text{=0}\text{.}1R$ and $R\text{=0}\text{.}1L$, which corresponds to the site width 0.7nm, the maximal number of sites is $(L-2R)/a\text{=80}$. It shows that the transport through the channel is low unless the sites are dense so that they occupy almost all the length of the pore. However, in this limit the transport is *sensitive* to the number of the binding sites, as illustrated in Fig. S3 B, which shows the ratio of the transport efficiency for the halved number of sites $n\text{/2}$, to that for $n$ sites. The transport efficiency is significantly reduced when the number of binding sites is large - i.e., in the regime of efficient transport. Therefore, the model with the inflexible FG-repeat regions is not capable to explain the observed robustness of the NPC transport with respect to the deletion of up to half of the mass of the FG-repeats (Strawn et al., 2004).

**High local occupancy of the channel.**

The maximal number of complexes that the channel can contain locally, depends on the ratio of the size of a karyopherin-cargo complex to the effective diameter of the channel. The size of a typical karyopherin is $\text{\textasciitilde}6\text{nm}\times6\text{nm}\times\text{10}\text{nm}$(Conti et al., 1998). Thus it is physically impossible that more than five karyopherins-cargo complexes occupy the same position along the channel; this number is even less in the case of large cargoes. Hence, in order to account for the possibility of several complexes present at the same position along the channel, we performed the simulations with the maximal local allowed occupancy $n_{\text{max}}\text{=5}$. The results of our simulations are shown in Fig. S4; these show that the transmission probability for $n_{\text{max}}\text{=5}$ does not substantially change compared to $n_{\text{max}}\text{=1}$.

**Fig. S1: Several binding sites on a single FG-repeat region result in an effective potential** Schematic representation of several binding sites on an FG-repeat region. Each black well corresponds to the diffusion of the karyopherin-cargo complex while bound to a particular binding site. As explained in the text, diffusion in the array of potentials of this type is equivalent to the diffusion in the effective potential shown in red line.

**Fig. S2: Array of discrete bindings sites** Array of narrow wells represents a situation where the FG-repeat regions are not flexible, and the binding sites are discrete and stationary.

**Fig. S3: Transport efficiency in the case of stationary binding sites.** **A**: Transport efficiency as a function of the number of discrete and narrow binding sites; the width of each site is $a\text{=0}\text{.}1R\text{\textasciitilde}0\text{.}7\text{nm}$, and its binding strength is $E\text{=10}k_{B}T$. The transport efficiency is low unless the sites are dense, so that sum of their lengths approaches the total length of the channel. **B**: Ratio of the transport efficiency for the halved number of sites, $n\text{/2}$ to that for $n$ sites, as a function of the number of sites $n$. In the region of high transport efficiency (large $n$), it is significantly reduced by halving the number of sites.

**Fig. S4: Karyopherins efficiently exclude non-specifically binding macromolecules from the NPC, irrespective of the permitted local occupancies.** Shown is the transport efficiency of particles across the NPC as a function of interaction strength with the FG-repeat regions, either in the presence or absence of competing particles. Gray line: transport efficiency of particles as a function of interaction strength in the absence of competition. *Red line*: transport efficiency of a weakly-binding species in an equal mixture of weakly- and strongly- binding species, as a function of the interaction strength of the weakly-binding species, in the case when the maximum allowed local occupancy is one particle; the interaction strength for the strongly binding species is $\text{12}k_{B}T$. *Blue line*: translocation probability of the weakly binding species in the case when the maximum allowed local occupancy is five particles. No RanGTP activity is included in these simulations.

**Fig. S5: Translocation probability for high local occupancy.** The black line shows the translocation probability in the case when only one complex can be present at any position along the channel; this models large cargoes. The red line is the translocation probability for the case when the channel can locally hold up to five complexes. The two curves do not differ substantially.
