# Supplementary material for: Efficiency, Selectivity, and Robustness of Nucleocytoplasmic Transport
Source: PLoS Comput Biol. 2007 Jul 13;3(7):e125. doi: 10.1371/journal.pcbi.0030125 (PMC1914370; doi:10.1371/journal.pcbi.0030125)
Supplement: Supplementary file 2 [file pcbi.0030125.sd002.doc]

**Supplementary text 2:** **Simulation code for the diffusion of complexes through the NPC channel**

/* Simulations code for:

"Efficiency, selectivity and robustness of the nuclear pore complex transport"

A. Zilman, S. Di Talia, B. Chait, M. Rout and M. Magnasco. */

/* This code implements the simulation of the diffusion of complexes through the NPC channel which is represented as an array of potential wells*/

/*The simulations rely on the Gillespie algorithm*/

/* The details of the algorithm are described in the text*/

#include<stdio.h>

#include<math.h>

#include<time.h>

#define LENGTH (1<<23) /*this parameter specifies the time length of the simulation */

#define capacity 5 /*this parameter specifies the maximum number of particles allowed at the same position*/

/*random number generator code starts here

(kindly donated by A. de Candia, Dipartimento di Scienze Fisiche, Universita' "Federico II, Naples, Italy) */

static int A[4];

static int B[4]={6698,7535,26792,30140};

/* 246913578*(2^32+1)=1060485742695258666 */

#define M0	17917	/* 13^13=302875106592253 */

#define M1	13895

#define M2	19930

#define M3	8

#define PW2 32767	/* 2^(15)-1 */

#define rpw2a 9.31322574615478516e-10	/* 2^(-30) */

#define rpw2b 1.11022302462515654e-16	/* 2^(-53) */

#define rpw2c 2.77555756156289135e-17	/* 2^(-55) */

double Xrandom(void)

{

A[0]=B[0]*M0;

A[1]=(A[0]>>15)+B[1]*M0+B[0]*M1;

A[2]=(A[1]>>15)+B[2]*M0+B[1]*M1+B[0]*M2;

A[3]=(A[2]>>15)+B[3]*M0+B[2]*M1+B[1]*M2+B[0]*M3;

B[0]=A[0]&PW2;

B[1]=A[1]&PW2;

B[2]=A[2]&PW2;

B[3]=A[3]&PW2;

return rpw2a*((B[3]<<15)+B[2])+rpw2b*((B[1]<<8)+(B[0]>>7))+rpw2c;

}

void init_random(int seed)

{

if (seed==0) seed=(int)time(NULL);

B[0]=(2+(seed<<2))&PW2;

B[1]=(seed>>13)&PW2;

B[2]=(seed>>28)&PW2;

B[3]=0;

Xrandom();

}

/*random number generator code ends here*/

/* this function is used to impose the condition that the maximum number of particle allowed at the same site is the capacity parameter*/

double sign (int x, int y) {

if ( x+y < capacity) return 1;

if (x+y >= capacity) return 0;

}

/* specify output file name, which includes the paremeters of the simulation*/

void nomefile1(double rate, int n, int length,int off,int cap, double ran, char *nome1)

{

sprintf(nome1, "selran=%lf_wells=%d_length=%d_off=%d_rate=%lf_cap=%d",ran,n,length,off,rate,cap);

}

int main(){

int k,i,j,l,wells,period,L,x0,indiceran,npotx,npoty;

double ax,ay,ratex,ratey,z,ran,t,flip,cumultot,Nfx, Nbx, Nfy, Nby;

char nome1[100];

FILE *file1;

/*set the channel parameters for the periodic potential*/

/*x0 is equivalent to the R, as described in the text, wells= number of potential wells, period= the number of sites in each well.*/

x0=4;

wells=1;

period=32;

L=wells*period;

/* define two arrays corresponding to the occupancies two particle types x and y which interact with potential U an V respectively*/

int x[L+2*x0+2], y[L+2*x0+2];

double Ux[L+2*x0+2],Uy[L+2*x0+2],pfx[L+2*x0+2],pbx[L+2*x0+2],pfy[L+2*x0+2],pby[L+2*x0+2],grates[4*(L+2*x0+1)],cumul[4*(L+2*x0+1)];

/*initialize the random number generator */

init_random(0);

for(indiceran=0;indiceran<1;indiceran++){ /*this is a loop over different values of ran (see paper for explanations)*/

ran=(double) 0.1*indiceran;

for(l=1;l<=1;l++){/* this is a loop over different entrance rates*/

ratex=(double)0.0001*l*50; /* entrance rate for particle of type x*/

ratey=0.0001; /*entrance rate for particle of type y */

/*define the name of the output file */

nomefile1(ratex,wells,L,x0,capacity,ran,nome1);

if((file1=fopen(nome1,"w"))==NULL)

{

printf("Unable to open file!\n");

}

/* set the potentials */

npoty=10; /*specify the interaction strength of potential Uy, for particles of type y */

/* it represents the specifically interacting particle */

for(npotx=0;npotx<11;npotx++){ /*loop over different values of potential Ux, for particles of type x*/

ax=(double) 4*npotx/((period)*(period));

ay=(double) 4*npoty/((period)*(period));

for(j=0;j<=x0;j++){

Ux[j]=0;

Uy[j]=0;

}

for(k=1;k<=wells;k++){

for(j=x0+1+(k-1)*(period);j<=k*(period)+x0-1;j++){

z=(double) j;

Ux[j]=(double) ax*(z-x0-(k-1)*(period))*(z-x0-k*(period));

Uy[j]=(double) ay*(z-x0-(k-1)*(period))*(z-x0-k*(period));

}

}

for(j=x0+L;j<=2*x0+L;j++){

Ux[j]=0;

Uy[j]=0;

}

/*jumping probabilities definitions*/

for(j=1;j<L+2*x0;j++){

pfx[j]=0.5*exp((Ux[j]-Ux[j+1])/2); /*probability to jump forward for particle x*/

pbx[j]=0.5*exp((Ux[j]-Ux[j-1])/2); /*probability to jump backward for particle x*/

pfy[j]=0.5*exp((Uy[j]-Uy[j+1])/2); /*probability to jump forward for particle y*/

pby[j]=0.5*exp((Uy[j]-Uy[j-1])/2); /*probability to jump backward for particle y*/

}

/* set initial conditions (in this case we choose an empty channel)*/

for(j=0;j<=L+2*x0;j++){

x[j]=0;

y[j]=0;

}

t=0;

Nfx=0; /* number of particle of type x getting to the nucleus */

Nbx=0; /* number of particle of type x going back to the cytoplasm */

Nby=0; /* number of particle of type y getting to the nucleus */

Nfy=0; /* number of particle of type y going back to the cytoplasm */

/* Compute the rates (grates), as described in the paper*/

grates[0]=ratex;

grates[4*(L+2*x0)-1]=ratey;

for(i=1;i<4*(L+2*x0)-1;i++){

grates[i]=0;

}

while(t<LENGTH){

for(i=1;i<L+2*x0;i++){

grates[i]=(double) pfx[i]*x[i]*sign(x[i+1],y[i+1]);

}

for(i=1;i<L+2*x0;i++){

grates[i+L+2*x0-1]=(double) pbx[i]*x[i]*sign(x[i-1],y[i-1]);

}

for(i=1;i<L+2*x0;i++){

grates[i+2*(L+2*x0)-2]=(double) pfy[i]*y[i]*sign(x[i+1],y[i+1]);

}

for(i=1;i<L+2*x0;i++){

grates[i+3*(L+2*x0)-3]=(double) pby[i]*y[i]*sign(x[i-1],y[i-1]);

}

grates[4*(L+2*x0)-3]=ran*x[L+x0];

grates[4*(L+2*x0)-2]=ran*y[L+x0];

for(i=0;i<=4*(L+2*x0)-1;i++){

cumul[i]=0;

}

for(i=0;i<=4*(L+2*x0)-1;i++){

for(j=0;j<=i;j++){

cumul[i]+=grates[j];

}

}

cumultot=cumul[4*(L+2*x0)-1];

for(i=0;i<=4*(L+2*x0)-1;i++){

cumul[i]= cumul[i]/cumultot;

}

/*draw a random number */

flip=Xrandom();

/*update the occupancies according to the according to the obtained random number, as explained in the text*/

if(flip<cumul[0]){

if(x[x0]+y[x0]<capacity){

x[x0]+=1;

}

else{

Nbx+=1;

}

}

else if(flip>cumul[4*(L+2*x0)-2]){

if(y[x0]+x[x0]<capacity){

y[x0]+=1;

}

else{

Nby+=1;

}

}

else if(flip<cumul[4*(L+2*x0)-4]){

for(i=1;i<=4*(L+2*x0)-4;i++){

if((flip>cumul[i-1]) && (flip<cumul[i])){

if(i<(L+2*x0-1)){

x[i+1]+=1;

x[i]=x[i]-1;

}

else if(i==(L+2*x0-1)){

Nfx+=1;

x[i]=x[i]-1;

}

else if (i<=2*(L+2*x0-1)){

j=i-(L+2*x0-1);

if(j==1){

Nbx+=1;

x[1]=x[1]-1;

}

else{

x[j-1]+=1;

x[j]=x[j]-1;

}

}

else if (i<3*(L+2*x0-1)){

j=i-2*(L+2*x0-1);

y[j+1]+=1;

y[j]=y[j]-1;

}

else if(i==3*(L+2*x0-1)){

j=i-2*(L+2*x0-1);

Nfy+=1;

y[j]=y[j]-1;

}

else{

j=i-3*(L+2*x0-1);

if(j==1){

Nby+=1;

y[1]=y[1]-1;

}

else{

y[j-1]+=1;

y[j]=y[j]-1;

}

}

}

}

}

else{

if(flip<cumul[4*(L+2*x0)-3]){

Nfx+=1;

x[L+x0]= x[L+x0]-1;

}

else{

Nfy+=1;

y[L+x0]= y[L+x0]-1;

}

}

/* advance time*/

t+=1/(cumultot)*log(1/Xrandom());

}

/*print the results into the output file*/

fprintf(file1, "%lf %lf Nfx= %lf pfx=%lf %lf Nfy=%lf pfy=%lf %lf\n",t,Ux[L/2+x0], Nfx, Nfx/(Nfx+Nbx), Nbx, Nfy, Nfy/(Nfy+Nby), Nby);

}

}

}

}
